# A competing risk analysis of sequential complication development in Asian type 2 diabetes mellitus patients

**DOI:** 10.1038/srep15687

**Published:** 2015-10-28

**Authors:** Li-Jen Cheng, Jeng-Huei Chen, Ming-Yen Lin, Li-Chia Chen, Chun-Huan Lao, Hsing Luh, Shang-Jyh Hwang

**Affiliations:** 1 Division of Nephrology, Department of Internal Medicine, Kaohsiung Medical University Hospital, Kaohsiung Medical University, Kaohsiung, Taiwan; 2 Department of Mathematical Sciences, National Chengchi University, Taipei, Taiwan; 3 National Applied Research Laboratories, Instrument Technology Research Center, Kaohsiung Medical University, Kaohsiung, Taiwan; 4 Division for Social Research in Medicines and Health, School of Pharmacy, University of Nottingham, Nottingham, UK; 5 Graduate Institute of Clinical Pharmacy, Kaohsiung Medical University, Kaohsiung, Taiwan; 6 Waikato Clinical School, The University of Auckland, Hamilton, New Zealand; 7 Faulty of Renal Care, College of Medicine, Kaohsiung Medical University, Kaohsiung, Taiwan; 8 Institute of Population Sciences, National Health Research Institute, Miaoli, Taiwan

## Abstract

This retrospective cohort study investigated the progression risk of sequential complication in Asian type 2 diabetes (T2D) patients using the Taiwan Pay-for-Performance Diabetes Registry and claim data from November 2003 to February 2009. 226,310 adult T2D patients without complication were followed from diagnosis to complications, including myocardial infarction (MI), other ischemic heart disease (IHD), congestive heart failure (CHF), stroke, chronic kidney disease (CKD), retinopathy, amputation, death or to the end of study. Cumulative incidences (CIs) of first and second complications were analyzed in 30 and 4 years using the cumulative incidence competing risk method. IHD (29.8%), CKD (24.5%) and stroke (16.0%) are the most common first complications. The further development of T2D complications depends on a patient’s existing complication profiles. Patients who initially developed cardiovascular complications had a higher risk (9.2% to 24.4%) of developing IHD or CKD, respectively. All-cause mortality was the most likely consequence for patients with a prior MI (12.0%), so as stroke in patients with a prior MI (10.8%) or IHD (8.9%). Patients with CKD had higher risk of developing IHD (16.3%), stroke (8.9%) and all-cause mortality (8.7%) than end-stage renal disease (4.0%). Following an amputation, patients had a considerable risk of all-cause mortality (42.1%).

Type 2 diabetes (T2D) is a common chronic disease characterised by progressive, interdependent but partially preventable complications, including cardiovascular diseases (CVDs), stroke, chronic kidney disease (CKD) and retinopathy^1^,^2^,^3^. Poorly-controlled diabetes is associated with a significantly increased morbidity and mortality resulting in an enormous healthcare burden^4^.

A rapid escalation of diabetes prevalence has been noted in Asian populations and this has become a global public health concern. The International Diabetes Federation predicts the number of people with diabetes worldwide will rise from 366 million in 2011 to 552 million by 2030, with half of this increase arising from Asia. Of the anticipated 186 million new diabetic cases, 39 million are expected from China and India, the two countries with the largest diabetic populations^5^. Consequently, provision of diabetic care presents particular challenges for this region^6^.

A range of evidence-based clinical guidelines^7^,^8^ have been developed to recommend optimal diabetes management strategies based on large randomised controlled trials, such as the Diabetes Control and Complications Trial^9^ and United Kingdom Prospective Diabetes Study^1^, and several epidemiological studies also inform risks of long-term complication in diabetes^1^,^2^. However, epidemiological data studying the development of complications in Asian patients with diabetes are limited.

Sub-group analysis in several Western studies suggested that key diabetes complications in Asian ethnicity differ from their Western counterparts^10^, for example, Asian diabetic patients have lower risks of CVDs and amputation^11^,^12^,^13^,^14^, but are more likely to develop nephropathy^15^,^16^,^17^ and retinopathy^18^. An improved understanding of these differences in susceptibility to develop complications may contribute to the development of effective screening and treatment strategies and lead to improvements in quality of care Asian diabetic patients^19^.

In addition, previous epidemiological research in quantifying the risk of diabetes-associated complications mostly reported the risk of first complication and assessed individual complications in isolation without considering patients’ complications profiles^11^,^12^,^13^,^14^,^20^ However, patients who have developed a complication, may consequently be more likely to develop another specific complication^7^.

A competing risk is an event that may hinder the observation of a clinical event or that may modify the chance for the occurrence of event. Therefore, a competing risk analysis incorporating sequential complications may be a better alternative to mostly used standard survival analysis methods, i.e. the Kaplan-Meier (KM) estimates and Cox regression. Indeed, Abbasi *et al.* (2013) who developed a risk prediction model of diabetes complications from literature also suggested that current analysis of diabetic progression of complications (without considering competing risk) greatly obscures the estimate on risk of complications across patients with various disease profiles^20^.

This study aimed to investigate the development of complications in T2D patients who have no complication at the diagnosis of diabetes using a longitudinal population-based database. The long-term cumulative incidence (CIs) of first and second major complications after the diagnosis of diabetes was analysed considering each complication as a competing risk to another within a single analytic framework rather than treating each clinical event separately, which encompasses the interactions between complications in estimating the risk of diabetes progression^21^,^22^,^23^.

## Results

### Characteristics of patients

Of all, 226,310 T2D patients (1,882,341 patient-years, median: 6.7 years) were included, 51.0% were female. The mean age of diagnosis was 53.0 ± 11.9 (range: 18.5, 98.1) and the mean duration of diabetes was 5.6 ± 6.3 (range: 0, 61.5) years. At baseline, the cohort’s mean haemoglobin A1c (HbA1c) level was 8.5 ± 2.10% (69.4 ± 23.1 mmol/mol); mean body mass index (25.8 ± 4.0 kg/m^2^), fasting triglycerides (176.6 ± 175.5 mg/dl) and low-density-lipoprotein cholesterol (119.1 ± 35.3 mg/dl) were slightly higher than normal; while mean systolic (133.65 ± 18.16 mmHg) and diastolic (79.9 ± 10.8 mmHg) blood pressures were within normal range (Appendix 1).

### Diabetic complications

**Figure 1:**
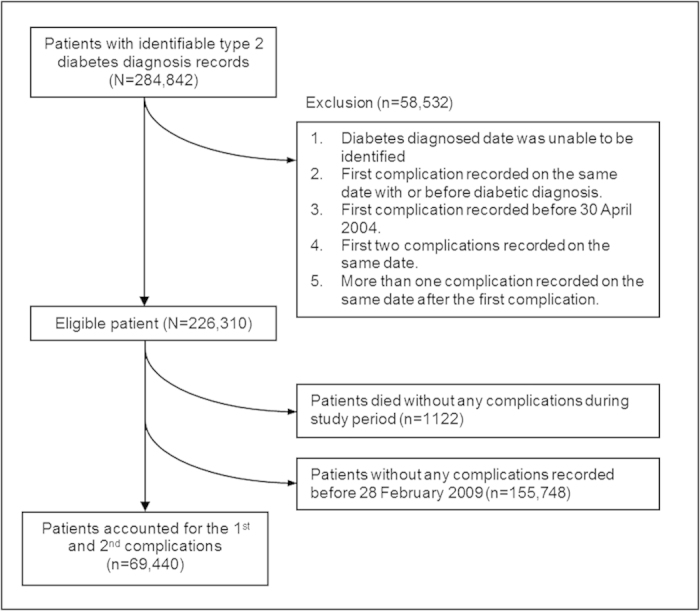
Process of identifying eligible patients in analysis.

Overall, 69,440 (30.7%) patients had developed at least one incidence of complication identified during the study period (Fig. 1). The total number of first and second complications identified including 2,702 myocardial infarction (MI), 33,319 other ischemic heart disease (IHD), 5,197 congestive heart failure (CHF), 17,176 stroke, 24,410 CKD, 38 amputation, 2,877 laser photocoagulation, 663 treatments for detached retina or vitreous haemorrhage and 4,264 (1,122 first and 3,142 second complications) all-cause mortality. In addition, recurrent events including 21 MI, 2,924 IHD, 297 CHF, 186 strokes and 617 end-stage renal diseases (ESRD) were identified at the second stage (Table 1).

### Cumulative incidence of the first complication

**Figure 2:**
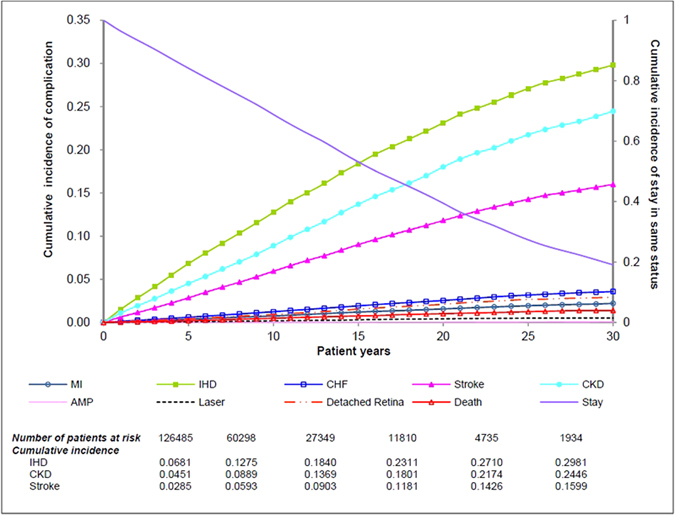
Cumulative incidence of the first diabetic complication over 30 years. (Note) MI: myocardial infarction; IHD: ischaemic heart disease; CHF: congestive heart failure; CKD: chronic kidney disease; AMP: amputation; stay: remaining no complication.

The 30-year cumulative incidences of first IHD, CKD and stroke (at the first stage) were found to be higher than other complications in all possible transitions after the diagnosis of diabetes (Fig. 2). The annual incidence of IHD, CKD and stroke ranged between 0.3% and 1.4%, resulting in a cumulative incidence of 6.8%; 4.5% and 2.9% after 5 years; 12.8%, 8.9% and 5.9% after 10 years; 23.1%; 18.0% and 11.8% after 20 years; and 29.8%, 24.5% and 16.0% respectively after 30 years. In contrast, the 30-year cumulative incidence of CHF, MI, retinopathy, amputation and all-cause mortality were lower than 4.0%.

Overall, 45,311 of the 69,440 (65.3%) patients only developed one complication during the study period. The proportions of patients developed a further complication varied and ranged from 25.1% (570/2272) in patients who developed retinopathy as the first complication and received laser photocoagulation, to 57.4% (1677/2923) in patients who developed CHF as their first complication (Table 2).

### Cumulative incidence of the second complication

Of the 24,129 patients who experienced more than one complication, patients with a prior CVD (MI, IHD, CHF and stroke) as the first complication, the 4-year cumulative incidence of progression was higher to IHD and CKD but lower to MI, retinopathy and amputation (Table 2). For instance, for patients with a prior CHF, the 4-year cumulative incidence of progression was higher to IHD (24.4%) and CKD (14.7%) but lower to MI (1.6%) and retinopathy (0.4% and 0.1%). In particular, the 4-year cumulative incidences of progressing to IHD were markedly high in patients with CHF (24.4%) and stroke (18.8%) and the incidences of progressing to stroke were also noticeably high in patients with MI (10.8%) and IHD (8.9%).

**Figure 3:**
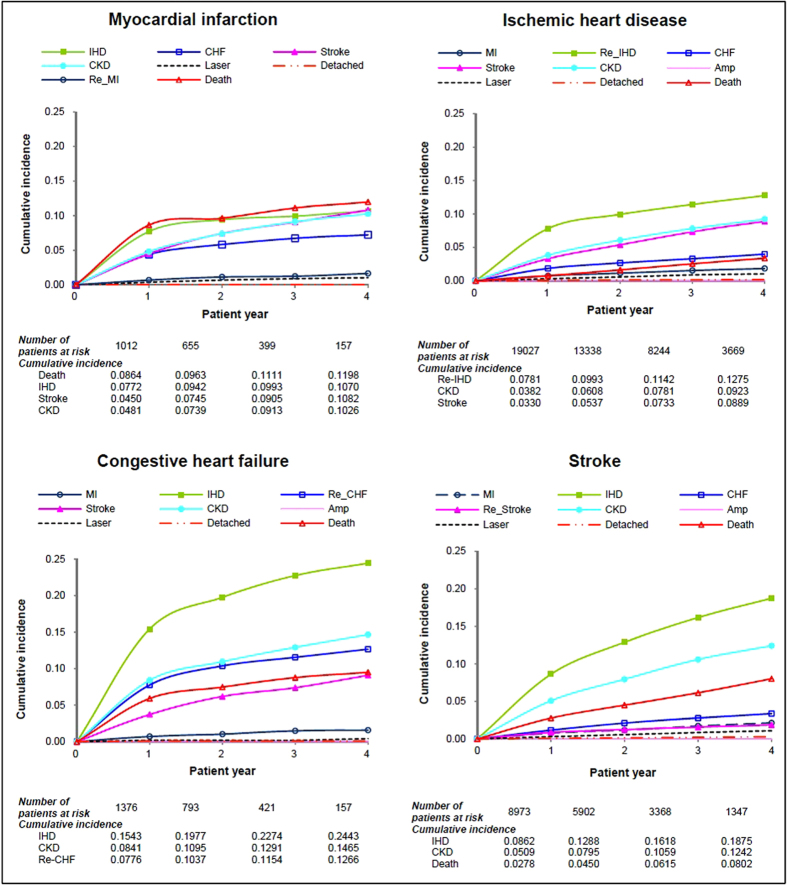
Four-year cumulative incidence of the second diabetic complication in patients with cardiovascular complications.

The 4-year incidence of recurrent CVDs (Fig. 3) was higher in patients with a prior IHD (12.8%) and CHF (12.7%) than patients with a prior MI (1.6%) and stroke (1.9%). All-cause mortality was the most likely consequence for patients with a prior MI (4-year incidence 12.0%) and it was also one of the largest threats for patients with a prior CHF (9.5%) and stroke (8.0%). It is also noteworthy that patients with a prior CHF had the lowest likelihood for staying without progression (4-year incidence 27.6%) comparing to patients with other cardiovascular complications.

**Figure 4:**
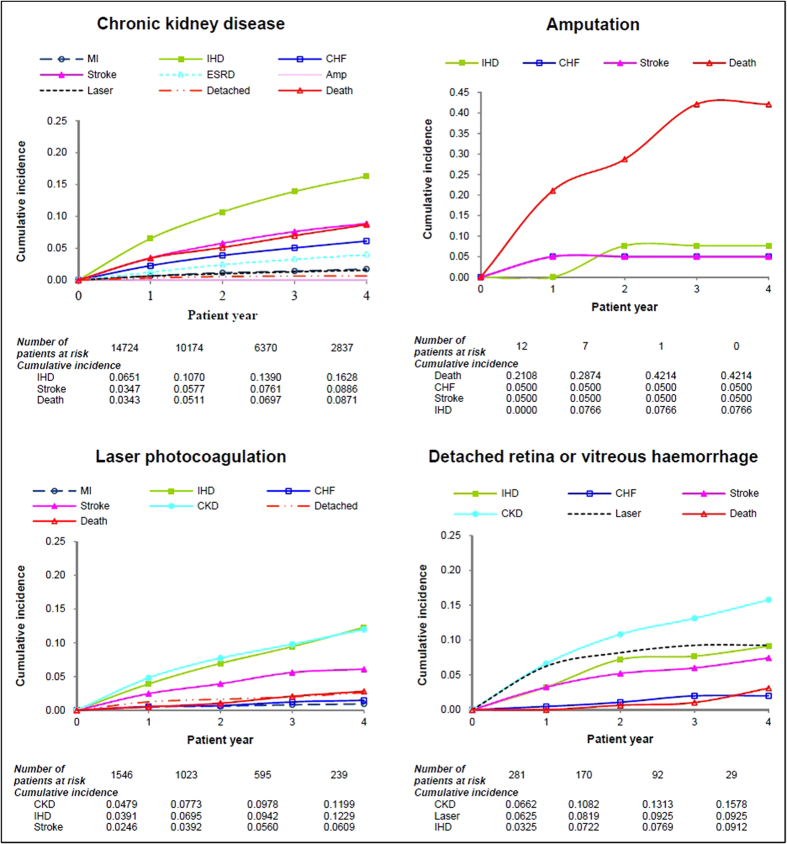
Four-year cumulative incidence of the second diabetic complication in patients with nephropathy, retinopathy and amputation.

For diabetic patients with a prior CKD, the 4-year cumulative incidences of progression to IHD (16.3%), stroke (8.9%) and all-cause death (8.7%) were higher than other complications (Fig. 4). Although ESRD is regarded as the renal complication that is likely to develop in patients with a prior CKD, the risk of progression to ESRD (3.98%) in patients with a prior CKD was lower than the risk of progression to other CVDs.

For diabetic retinopathy patients who either received laser photocoagulation or treatments for detached retina or vitreous haemorrhage, the 4-year cumulative incidence of progression was high to CKD (12.0%; 15.8%) and IHD (12.3%; 9.1%); moreover, the 4-year cumulative incidence of receiving laser photocoagulation for a new lesion was appreciable (9.3%). Finally, diabetic patients who received amputation had a considerable 4-year cumulative all-cause mortality of 42.1%.

## Discussion

This study investigated the progressive development of diabetes complications in a large cohort of Asian Chinese T2D patients by modelling the first and second major complications in a single analytical framework. Adult patients enrolled in the Taiwan Diabetes Pay-for-Performance (PFP) Registry were more likely to develop IHD, CKD and stroke than to develop MI, CHF, retinopathy and amputation as the first complication that need emergency or inpatient care. In addition, we found the risk of developing a second complication varies by patients’ existing complication profiles prior to the occurrence of further complications.

For patients with a prior CVD (MI, IHD, CHF and stroke), the risk of developing a further IHD and CKD was markedly higher than other complications, and stroke was one of the major complications for diabetic patients with a previous MI. Diabetic patients with CKD faced a higher risk of progressing to CVDs and all-cause mortality than progressing to ESRD within 4 years posterior to the diagnosis of CKD. The presence of retinopathy also contributed to an increased risk of impairing renal function. These results are expected based on previous epidemiological studies^24^,^25^,^26^.

Previous studies that have examined the risks of multiple diabetic complications mostly only reported the first occurrence of MI, CHF, stroke, ESRD and lower-extremity amputation (LEA) after diagnosis of diabetes as age- and sex-adjusted number of events per 1,000 patient-years. These studies also compared complication risks among different ethnic subgroups (Chinese and Asian patients) with whites in Canada^13^,^14^ and the United States^11^,^12^. In general, these studies showed the incidence of developing CVDs was significantly higher than the incidence of developing ESRD and LEA in Chinese diabetic patients.

For example, studies using the Kaiser Permanente Northern California Diabetes Registry^11^,^12^ and Canadian government-funded insurance programme in Ontario^14^ found that the risk of stroke (3.0–7.2/1000 person-year) was lower than MI (3.6–9.3/1000 person-year) during a 8-year study period (median follow up was 4.7 years) but slightly larger than CHF (2.5–5.9 /1000 person-year) for Chinese diabetes patients resident in Western countries, and this is different from the risk of the first complication estimated in our study.

However, another Canadian study using the administrative data from British Columbia and Alberta found that the risk of stroke (4.1–4.2/1000 person-year) is higher than MI (1.5–2.8/1000 person-year) and CHF (2.3–2.0/1000 person-year)^13^ for Chinese patients, which is similar to our findings. The discrepancies in complication risks reported for Chinese diabetic patients residing in different areas may be due to the variations in research settings, sample sizes, criteria used to identify complications and follow-up time, and hence warrants further exploration.

The previous studies^11^,^12^,^13^,^14^ also reported that white diabetes patients had a significantly higher risk of developing CVDs as the first complication than ESRD and LEA; of the CVDs, the highest risk was MI, and the risks of stroke and CHF were approximately equivalent. Comparing with these findings, our study also demonstrated the risk of amputation was the lowest among diabetic complications. However, our patients were at relatively lower risk to develop CHF and MI, instead more likely to develop the non-fatal coronary heart disease (i.e. IHD) as the first complication.

It has been widely reported in the literature that for the first complication occurring in Asian diabetes patients, the prevalence of diabetic nephropathy was higher^15^,^17^ and there was a lower prevalence of ESRD than CVDs. However, there is a paucity of data on comparing the risk of CKD with other diabetic complications in both Asians and whites. In our analysis, the risk of CKD as the first complication was higher than stroke, CHF, MI and retinopathy; and diabetic patients with CKD were at a lower risk of developing further ESRD than CVDs, except for MI.

This study also identified a high risk of CKD in patients with existing CVDs. Although the effect of prevalent CVD on developing a further kidney disease has been poorly investigated in prior reports, our findings are comparable to the results of a large community-based cohort study, which examined the association between CVD and subsequent kidney function decline and development of kidney disease^27^. Several CKD prediction models have also shown that CVD, stroke and CHF were the risk factors for the occurrence of CKD^28^,^29^,^30^.

The observations listed above, together with the noticeable risk of CKD after retinopathy, suggests that diabetic patients with either micro- or macro-vascular complication in this study were inclined to develop CKD, thereby threatening to cause fatal or disabling events, with a significant potential for progressing to ESRD or another cardiovascular complication. Early identification of CKD allows the best opportunity to initiate therapy to reduce renal risk factors and decelerate the loss of kidney function^31^, therefore, routinely monitoring kidney function should be recommended for diabetic patients in these high-risk groups. At present, routine kidney function monitoring (serum creatinine and urine albumin-to-creatinine ratio every 3 to 6 months) is recommended by the Taiwan Society of Nephrology and diabetologists are also encouraged to refer diabetic patients to the Taiwan National Health Insurance (NHI) early-CKD and pre-ESRD PFP Programmes.

The risk of all-cause mortality for patients who received amputation as the first complication after diabetes was as high as 42.14%. A number of studies have shown a high mortality (about 50% at 3 years) following amputation of a limb in patients with various diabetes complications^32^,^33^. However, it is surprised to observe this in patients who had no other complication except for diabetic foots. We hypothesise this result could be associated with patients’ socioeconomic background or inhabitant in remote area, as patients did not access any healthcare before diabetes progressed. Further studies should be carried out to test this hypothesis when relevant information is available.

To our knowledge, this study is one of the largest study populations with the longest follow-up published to date on disease progression of Asian diabetic patients. The large number of study cohort and the comprehensive PFP medical records increase the validity of identifying relevant clinical outcomes in diabetes progression. This study is also the first to investigate changes in the type and number of major diabetic complications within a single analytic framework. It also comprehensively captured different pathways in which diabetic complications or composite complications occurred, and thereby enabled identifying the most likely events among all possible increased risks after diabetes.

However, this study has several limitations. First, the baseline clinical indicators in the PFP Diabetes Registry were collected after November 2003, which varied for individuals at different stages of disease progression, and therefore clinical indicators (e.g. laboratory data) were not appropriate for adjustment. In addition, this study aimed to quantify the long-term cumulative incidence of first and second major complications after diabetes rather than compare results across different subgroups. Moreover, the results are consistent after adjusting for age and gender in a single-cause competing risk model; hence the unadjusted results were reported.

Second, the selection bias is acknowledged as contracted physicians have the discretion to enrol patients in an incentivised payment scheme. Previous studies also have indicated diabetic patients enrolled in the PFP scheme were healthier than those who were not recruited^34^,^35^. This study also found the cumulative incidences of complications were relatively low. Therefore, it should be cautious in applying the observed relation between sequential complications to diabetic patients with poor health.

Third, for patients who were diagnosed as diabetic before enrolment or November 2003, the self-reported diagnosed year cannot be confirmed and this may have caused recall bias and obscured some observed disease progression. Finally, there was no information on the medication and treatment of patients before November 2003, and hence it is unclear whether some patients received amputation or any treatment for retinopathy during this period.

The progression of diabetes in Asian patients is different from that reported in western countries. IHD, CKD and stroke are more likely the first complication than CHF, MI, retinopathy and amputation for Asian patients. Risk for developing a further complication varies according to patients’ existing complication profiles, and patients with an existing cardiovascular complication or retinopathy have a higher risk of developing IHD and CKD. These results inform the clinical decisions on prioritising monitoring and interventions for Asian diabetic patients who are at high risk of developing severe complications in this region.

## Methods

### Study design and data source

This retrospective cohort study was conducted using the Taiwan PFP Diabetes Registry from November 2003 to February 2009 after being granted approval from the Department of Health (DOH97-NH-1017) in Taiwan. The study protocol was also approved by the Institutional Review Board of Kaohsiung Medical University Hospital (KMUH-IRB-EXEMPT-20130038). All research procedures followed the directives of the Declaration of Helsinki.

The PFP Programme for Diabetes in Taiwan is one of the nationwide PFP programmes implemented since 2001^36^. It incentivises healthcare providers to deliver comprehensive monitoring and follow-up care by additional bonus payment. Contracted physicians and hospitals participate in the programme on a voluntary basis and have the discretion to recruit patients into the programme^35^.

The PFP Diabetes Registry contains detail information of registered diabetes patients, such as demography (age, gender), past medical history, diabetes-specific physical examinations, and laboratory data recorded at each follow-up visit. In addition, the PFP Diabetes Registry also links to the full NHI claim database of registered diabetes patients, such as outpatient and inpatient medical claims, and dispensing claims from community pharmacies, thus allows a valid estimate of treatment outcomes measures in this longitudinal follow-up study.

All the NHI claim data and PFP Diabetes Registry data are anonymous and detailed description of the regular NHI claim database has been reported elsewhere^37^. The PFP database together with NHI claim database provides rich data to investigate patients’ diabetic progression.

### Study cohort

Adults (aged 18 and above) with either incident or prevalent T2D who met the inclusion criteria: (1) were enrolled in the PFP programme before February 2009, (2) diabetes diagnosed date (disease index date) was identifiable, and (3) had no recorded diabetic complication before diagnosed date were included in this study. Patients’ medical claim data between November 2003 and February 2009 were extracted from the NHI claim database. To ensure the included patients had no diabetic compilation, those who had any diabetic complications recorded before 30 April 2004 (i.e. 6 months after beginning of follow-up) were excluded from this study.

Patients’ diabetes index dates were identified based on records in the PFP Registry. For patients whose diabetes was diagnosed before their enrolment into the programme or before November 2003, only ‘diagnosed year’ is available in the Registry, and hence June 30th of the year was used as the index date for those patients. The identified cohort was followed from individual diabetes index date to complications, death or the end of study, whichever occurred first.

### Outcome measure

The primary outcomes of this study were incidences of eight diabetes-related complications during the follow-up period, including MI, IHD (excluding MI), CHF, stroke, CKD and ESRD, retinopathy (defined as receiving laser and vitreoretinal surgery), amputation, and all-cause mortality, that required inpatient or emergency care.

The outcome events and event dates were identified by screening the corresponding International Classification of Diseases, Ninth Revision (ICD-9) diagnosis or procedure codes^38^ and therapeutic medicines recorded in the NHI claim database for emergency or inpatient care (Appendix 2), and several exclusion criteria were applied to ensure the occurrence of incidences (Table 3). For a mortality event, the mortality date was retrieved from the Registry, otherwise it was defined as three months after the patient’s last record in the NHI claim database as it is unusual that a diabetic patient will not access NHI healthcare in Taiwan^39^.

### Data analysis

**Figure 5:**
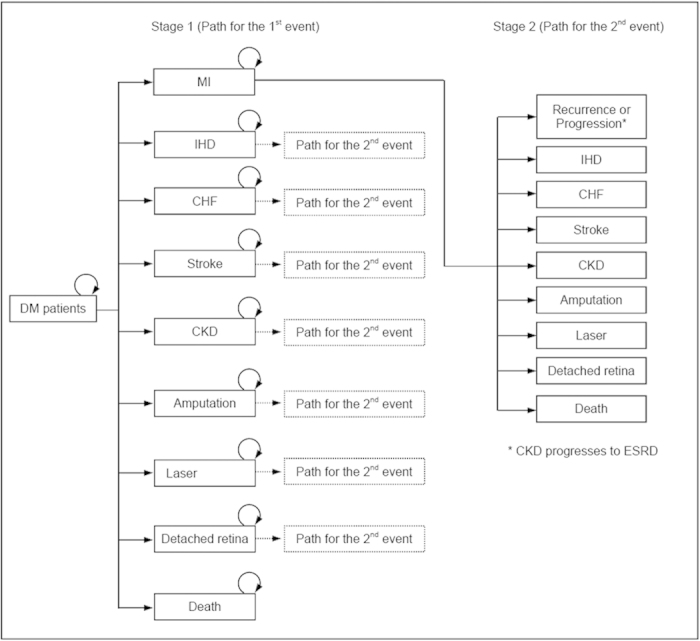
Conceptual framework for the pathway of first two events of diabetic complications. (Note) DM: diabetes mellitus; MI: myocardial infarction; IHD: ischemic heart disease; CHF: congestive heart failure; CKD: chronic kidney disease; ESRD: End Stage Renal Disease; Laser: patients received laser photocoagulation; Detached retina: patients received treatments for detached retina or vitreous haemorrhage.

All outcome events for individual patient were chronologically sequenced according to the event dates. The analysis focused on two disease progression stages, including: (1) the first stage—patients progressed from no complication to the first outcome incident occurring and (2) the second stage—patients progressed after the first event to when the second outcome event occurred. The observation end point in the first stage was any incidence of MI, IHD, CHF, stroke, CKD, amputation, receiving laser and vitreoretinal surgery and all-cause mortality. In the second stage, ESRD and recurrence (i.e. any repeat episode need inpatient or emergency care) of MI, IHD, CHF and stroke were recorded as the progression of cardiovascular and renal complications, instead of incident events (Fig. 5).

Event and time-to-event were calculated for each stage, and patients who remained in a same event stage were followed to the end of study. Descriptive statistics were used to report enrolled patients’ demographic and clinical characteristics. Cumulative incidences of the first events over 30 years and the second events over 4 years were calculated using the Kaplan-Meier cumulative incidence competing risk method. All data were processed using SAS software, version 9.2 (SAS Institute Inc.). The competing risk analysis was conducted by using R statistical programme, version 3.0.2, with the cmprsk package.

## Additional Information

**How to cite this article**: Cheng, L.-J. *et al.* A competing risk analysis of sequential complication development in Asian type 2 diabetes mellitus patients. *Sci. Rep.* **5**, 15687; doi: 10.1038/srep15687 (2015).

## Supplementary Material

Supplementary Information

## Figures and Tables

**Table 1:** Number of the first and second event of complications identified.

	First complication	Second complication	Total
**Myocardial infarction**	1,801	901	2702
**Ischemic heart disease**	27,859	5460	33319
**Congestive heart failure**	2,923	2274	5197
**Stroke**	13,513	3663	17176
**Chronic kidney disease**	20,605	3805	24410
**Amputation**	23	15	38
**Laser photocoagulation**	2,272	605	2877
**Treatments for detached retina or vitreous haemorrhage**	444	219	663
**All-cause mortality**	1,122	3142	4264

**Table 2:**
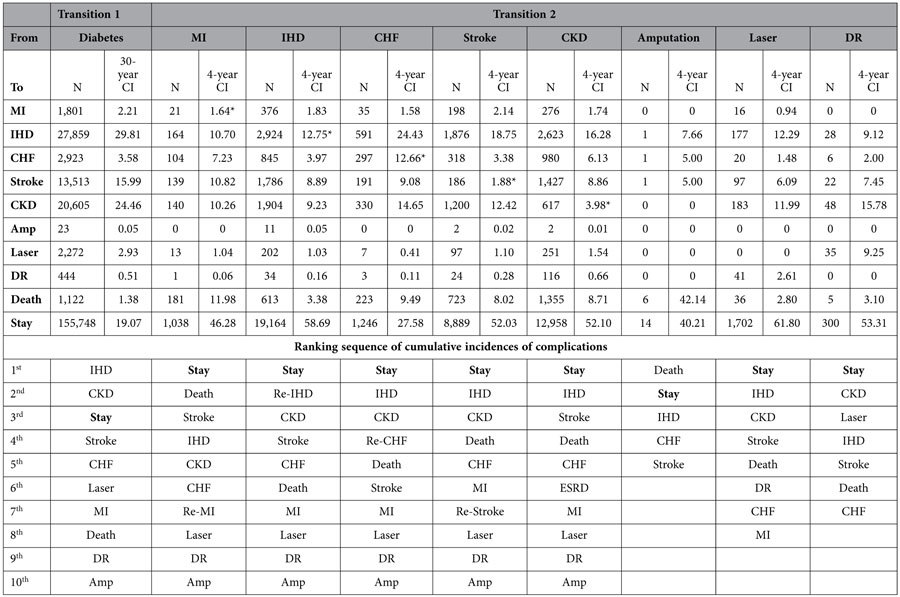
Cumulative incidences of the first and second event of complications.

(Note) N: number of patients; CI: cumulative incidence (%); Amp: amputation; Laser: the status that patients received laser photocoagulation; DR: detached retina denotes the status that patients received treatments for detached retina or vitreous haemorrhage; Stay: patients did not progress to another status; *ESRD and the recurrence of MI, IHD, CHF and stroke.

**Table 3:** Inclusion and exclusion criteria in outcome definitions.

Outcomes	Inclusion criteria			Exclusion criteria
	*ICD-9 diagnosis codes*	*Therapeutic drug codes or ICD-9 procedure codes*	*Source dataset for identifying records*	
Ischemic heart disease	411–414 or 4400–4402	drug codes	emergency or inpatient	14 days before or after a MI event
Congestive heart failure	428	drug codes	emergency or inpatient	14 days before or after a MI event
Myocardial infarction	410 or 412	drug codes	emergency record and followed by inpatient record within 30 days	
Stroke	430–438	drug codes	emergency record and followed by inpatient record within 30 days	
Chronic kidney disease	016.0, 095.4, 189.0, 189.9, 236.91, 274.1, 283.11, 403.×1, 404.×2, 404.×3, 440.1, 442.1, 447.3, 572.4, 580–588, 591, 642.1, 646.2, 753.12–753.17, 753.19 and 753.2		at least twice in the outpatient dataset or once in inpatient record within a year	
End-stage renal disease	Same as chronic kidney disease	Procedure codes of peritoneal dialysis or haemodialysis; drug codes of renal transplantation	haemodialysis more than 40 times in three consecutive months	
Laser photocoagulation		60005C or 60006C		7 days before or after a treatment for detached retina or vitreous haemorrhage
Detached retina	3619	85608B, 86403B and 86404B		
Vitreous haemorrhage	37923	86206B		
Amputation	Diabetic foot ulcer (707.10) or peripheral arterial occlusive disease (444.22)	V497		
Death		Classified as death in the Registry or no medical record for more than 6 months before February 2009		
