# Supplementary material for: A competing risk analysis of sequential complication development in Asian type 2 diabetes mellitus patients
Source: Sci Rep. 2015 Oct 28;5:15687. doi: 10.1038/srep15687 (PMC4623532; doi:10.1038/srep15687)
Supplement: Supplementary Information [file srep15687-s1.pdf]

Supplement for the manuscript submitting to Scientific Report

Title

A competing risk analysis of sequential complication development in Asian type 2 diabetes mellitus patients

Authors

Li-Jen Cheng[1], Jeng-Huei Chen[2], Ming-Yen Lin[1,3], Li-Chia Chen[4,5], Chun-Huan Lao[6], Hsing Luh[2], Shang-Jyh Hwang[1,7,8]

Affiliations

[1] Division of Nephrology, Department of Internal Medicine, Kaohsiung Medical University Hospital, Kaohsiung Medical University, Kaohsiung, Taiwan

[2] Department of Mathematical Sciences, National Chengchi University, Taipei, Taiwan

[3] National Applied Research Laboratories, Instrument Technology Research Center, Kaohsiung Medical University, Kaohsiung, Taiwan

[4] Division for Social Research in Medicines and Health, School of Pharmacy, University of Nottingham, Nottingham, U.K.

[5] Graduate Institute of Clinical Pharmacy, Kaohsiung Medical University, Kaohsiung, Taiwan

[6] Waikato Clinical School, The University of Auckland, Hamilton, New Zealand

[7] Faulty of Renal Care, College of Medicine, Kaohsiung Medical University, Kaohsiung, Taiwan

[8] Institute of Population Sciences, National Health Research Institute, Miaoli, Taiwan

Corresponding author

Li-Chia Chen

Characteristics	Value	Missing value (%)
Total number of patients (%)	222,808 (100)	
Demography		
Number of female patients (%)	113,596 (50.98)	
Mean diagnosed age (year)	53.00±11.86	1 (0.00)
Mean entry age (year)	58.55±12.04	692 (0.31)
Number of patient in entry age ranks (%)		
≤35 years	7,478 (3.37)	
36-45 years	25,427 (11.45)	
46-55 years	61,751 (27.80)	
56-65 years	63,982 (28.81)	
66-75 years	46,995 (21.16)	
76-85 years	15,432 (6.95)	
≥86 years	1,051 (0.47)	
Disease history and follow-up duration		
Total follow-up duration (person-year)	1,882,341.04	
Mean follow-up duration (person-year)	8.45±6.54	
Mean DM history at entry (year)	5.56±6.28	693 (0.31)
Laboratory data		
Mean body mass index (kg/m2)	25.81±3.97	1271 (0.57)
Mean systolic blood pressure (mmHg)	133.65±18.16	591 (0.27)
Mean diastolic blood pressure (mmHg)	79.89±10.82	591 (0.27)
Mean HbA1c (mmol/mol; %)	69.4±23.1 (8.5±2.10%)	394 (0.18)
Mean HDL cholesterol (mg/dl)	47.10±13.09	91985 (41.28)
Mean LDL cholesterol (mg/dl)	119.05±35.28	91985 (41.28)
Mean Triglyceride (mg/dl)	176.64±175.51	3501 (1.57)

(Note) Overall, 3,502 patients did not have clinical information reported in the Registry, and therefore only the information of 222,808 patients is presented in the table. Delta refers to difference between systolic and diastolic blood pressure.

Appendix 1. Characteristics of the included type 2 diabetes patients

Indication	Classification	Drug name	ATC code
Myocardial infarction or ischemic heart disease	Fibrinolytic agent	Alteplase	B01AD02
		Tenecteplase	B01AD11
		Streptokinase	B06AA55, B01AD01
	Aspirin	Aspirin	B01AC06, N02BA01
	Thienopyridines	Clopidogrel	B01AC04
		Ticlopidine	B01AC05
	Glycoprotein IIb/IIIa receptor inhibitors	Abciximab	B01AC13
		Eptifibatide	B01AC16
		Tirofiban	B01AC17
	Anticoagulants		
	Unfractionated heparin (UFH)	Heparin	B01AB01
	Low-molecular-weight heparins (LMWHs)	Enoxaparin	B01AB05
		Dalteparin	B01AB04
	Nitrates	Nitroglycerin	C01DA02, C01DA52
	β-Adrenergic Blockers	Propranolol	C07AA05
		Atenolol	C07AB03
		Esmolol	C07AB09
Congestive heart failure	Loop diuretics	Furosemide	C03CA01
		Bumetanide	C03CA02
	Positive inotropic Agents	Dobutamine	C01CA07
		Milrinone	C01CE02
		Dopamine	C01CA04
	Vasodilators	Nitroprusside	C02DD01
		Nitroglycerin(glyceryl trinitrate)	C01DA02, C01DA52
Ischemic stroke	Fibrinolytic agent	Alteplase	B01AD02
	Aspirin	Aspirin	B01AC06, N02BA01
	Thienopyridines	Clopidogrel	B01AC04
	Anticoagulants	Warfarin	B01AA03

Appendix 2. Therapeutic drug codes and procedures code used to identify outcome events
